# Identification of a new amino acid mutation in the HN protein of NDV involved in pathogenicity

**DOI:** 10.1186/s13567-021-01019-4

**Published:** 2021-12-20

**Authors:** Xi Chen, Yanqing Jia, Ning Wei, Chao Ye, Huafang Hao, Sa Xiao, Xinglong Wang, Haijin Liu, Zengqi Yang

**Affiliations:** 1 College of Veterinary Medicine, Northwest A&F University, Yangling, 712100 Shaanxi China; 2 College of Animal & Veterinary Sciences, Southwest Minzu University, Chengdu, China; 3 Department of Animal Engineering, Yangling Vocational & Technical College, Yangling, 712100 Shaanxi China

**Keywords:** Newcastle disease virus, HN protein, amino acid mutation, chimeric viruses, membrane fusion, viral propagation, pathogenicity

## Abstract

The fusion (F) and haemagglutinin-neuraminidase (HN) proteins of Newcastle disease virus (NDV) are viral entry proteins and are recognized as the major virulence determinants. Previously, a lentogenic NDV virus (CE16) was derived from a mesogenic strain (CI10) through sequential passages in chick embryos. Whole-genome sequence analysis revealed that the two homologous strains shared the same F protein but differed in HN with two amino acid (aa) substitutions (A215G and T430A). To elucidate the molecular reasons for virulence attenuation, two original plasmids (HN-CI10 and HN-CE16) and two single-point mutants (G215A and A430T) reverse-mutated from HN-CE16 were constructed to analyse the known biological functions of HN. The results showed that the A430T substitution significantly weakened the haemadsorption (HAd) activity, increased the neuraminidase (NA) activity, improved the fusion-promoting activity, and enhanced the cleavage-promoting activity of HN-CE16. However, G215A failed to induce obvious functional changes. Therefore, the aa residue HN430 may play a key role in determining virulence. To test this hypothesis, further studies on A430T were conducted through reverse genetics using an infectious cDNA clone. At the viral level, the A430T-mutated virus showed dramatic promotion of viral plaque formation, propagation, and pathogenicity in vitro and in vivo. This study demonstrates a new virulence site associated with HN protein functions, viral propagation, and pathogenicity. All these findings could lay a foundation for illuminating the molecular mechanism of NDV virulence.

**Supplementary Information:**

The online version contains supplementary material available at 10.1186/s13567-021-01019-4.

## Introduction

Newcastle disease (ND) caused by Newcastle disease virus (NDV) is one of the most severe infectious diseases of birds and has caused serious economic losses in the poultry industry worldwide [1, 2]. NDV belongs to the genus *Orthoavulavirus* of the family *Paramyxovirinae* and has an enveloped, nonsegmented, negative-sense RNA genome [3]. The genome is approximately 15 kb and contains six open reading frames (ORFs) that encode the nucleoprotein (NP), phosphoprotein (P), matrix (M) protein, fusion (F) protein, haemagglutinin-neuraminidase (HN) protein, and large (L) protein [4].

NDV infection requires the fusion of the virion envelope with the host cell membrane, which is triggered by the cooperation of two surface glycoproteins, F and HN [5, 6]. The attachment of NDV to the surface of the target cell is initiated by binding of the viral HN to the sialic acid-containing receptor, which further induces conformational changes of the F protein to expose the cleavage site (FCS). Then, the F protein is cleaved from F0 into F1 and F2 by host cell proteases, and the fusion peptide of F1 inserts into the cell surface to initiate membrane fusion [7–9]. Usually, the molecular basis for NDV pathogenicity is mainly determined by the FCS. In general, the FCS of velogenic and mesogenic NDV is ^112^R/K-R-Q-R/K-R F^117^, and that of lentogenic viruses is ^112^G/E-K/R-Q-G/E-R L^117^ [10]. The HN protein is a multifunctional protein consisting of a globular head and a membrane-anchored stalk domain. The globular head has haemagglutinin (HA) activity, by which it recognizes and binds sialic acid-containing receptors on the host cell surface, and neuraminidase (NA) activity, by which it hydrolyses sialic acid to release progeny virions. Then, the stalk interacts with the F protein to promote membrane fusion [11–13].

Correlations between pathogenicity and HN biological activities have been observed in some reverse genetics studies upon mutating specific residues [14–16]. P93A- and L94A-bearing viruses display impaired receptor recognition ability, NA activity, and fusion-promoting activity, all of which lead to virus attenuation. In addition, an L94A-mutated virus shows a dramatic decline in replication [14]. The mutation Y526Q results in a decrease in viral HA activity, NA activity, and fusion activity and has an attenuating effect on growth kinetics in cell culture and pathogenicity [15]. R596C in the C-terminal extension of the HN protein results in a reduced level of HA, which contributes to virus attenuation [16].

Previous studies have reported the derivation of a lentogenic NDV virus from a mesogenic strain through sequential passages in chick embryos. Sequence analysis has revealed that the two homologous strains share the same F protein but differ in HN, with two aa substitutions [17]. Therefore, we hypothesized that these two aa sites may lead to changes in the functions of the HN protein that ultimately cause the difference in virulence between the two strains. This study was based on these two aa sites and was conducted to test the effects of HN mutations on the fusion ability, propagation, and pathogenicity of NDV. Elucidating these effects could help to identify key sites in the HN protein causing virulence differences and will provide a theoretical basis for exploring the molecular mechanism of NDV virulence.

## Materials and methods

### Cells, viruses, and animals

Chicken fibroblasts (DF-1), hamster kidney cells (BHK-21), and human epidermoid cancer cells (Hep-2) were grown in Dulbecco’s modified Eagle's medium (DMEM) (Gibco) supplemented with 10% foetal bovine serum (FBS), 100 U/mL penicillin and 100 mg/mL streptomycin. The crested ibis/China/10 (CI10) strain was isolated from a crested ibis population with Newcastle-like disease with an intracerebral pathogenicity index (ICPI) of 1.04. The chicken embryos/China/16 (CE16) strain was derived by the passage of CI10 through chicken embryos with an ICPI of 0.35 [16]. Both strains were plaque-purified three times consecutively in BHK-21 cells and stored in our laboratory. MVA/T7 expressing T7 RNA polymerase was kindly donated by Professor Siba K. Samal (University of Maryland, USA). Ten-day-old SPF chicken embryos and 1-day-old and 4-week-old chickens were purchased from Jinan SAIS Poultry Company (Shandong, China).

### Plasmid construction and site-directed mutagenesis

The CI10 and CE16 virus strains were used for viral RNA extraction according to the manufacturer’s protocol supplied with the StarSpin Animal RNA Mini Kit (Genstar, Beijing, China), and cDNA was synthesized from the viral RNA using a reverse transcription PCR kit (Genstar, Beijing, China). Specific primers (Additional file 1) for F and HN were used to amplify the ORFs of F and HNs using PrimerSTAR Max DNA Polymerase (TaKaRa, Beijing, China). The amplified HN genes were inserted into the eukaryotic expression vector plasmid pCAGGS containing EcoRI and XhoI restriction sites. The F gene was inserted into the vector pCAGGS containing ClaI and XhoI restriction sites, and an HA tag was added to the C-terminus of the F protein for detection. Site-directed mutagenesis was employed to produce the HN mutations G215A and A430T with specific primers (Additional file 1). Briefly, primers with single-point mutations were first designed. Then, using HN-CE16 as a template, the first fragment was amplified with the primer HN-F and the site-directed primer R, and the second fragment was amplified with the site-directed primer F and the primer HN-R. Then, the 2 fragments were used as templates for overlapping PCR, and the PCR products were inserted into the vector pCAGGS. The synthesis of primers and sequencing of these recombinant plasmids were performed by TSINGKE (Beijing, China).

### Virus recovery

**Figure 1:**
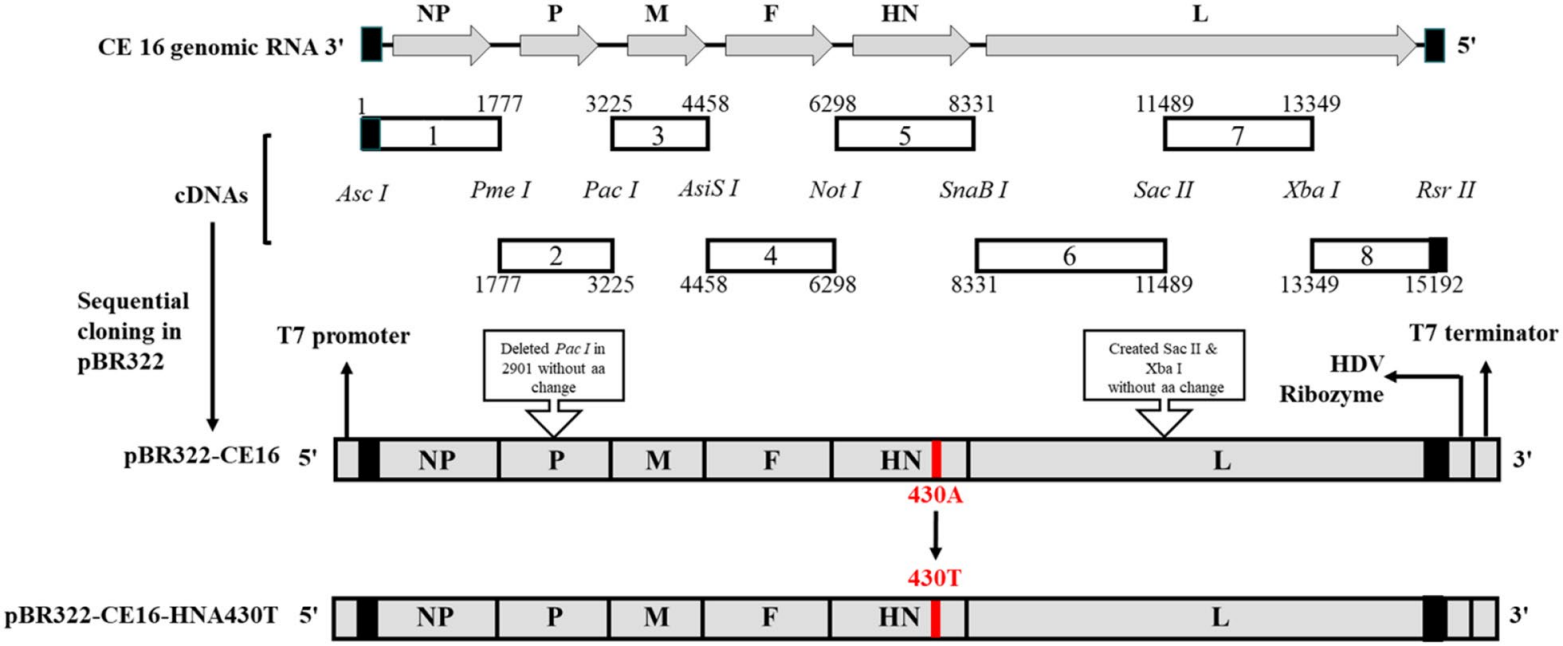
**Schematic representation showing the construction of pBR322-CE16 and pBR322-CE16-HNA430T.**

The NP, P, and L of CE16 were cloned into the pCAGGS vector to construct helper plasmids (Additional file 2). To generate the full-length antigenome, a total of eight pairs of primers were designed (Additional file 3) and used to produce cDNA fragments, which were then ligated stepwise into the pBR322 vector (pBR322-CE16) (Figure 1). Fragment 1 had a T7 promoter overhang after the *AscI* restriction site, and fragment 8 had a 24-nucleotide (nt) partial HDV ribozyme sequence overhang before *RsrII*. Additionally, the primers corresponding to fragment 2 were modified at nt positions 2903 and 2906 to delete the unnecessary *PacI* site, and the primers corresponding to fragments 6, 7, and 8 were modified at nt positions 11,494 and 13,354 to create *SacII* and *XbaI* site tags without aa changes. By using specific primers, A430T was introduced into HN-containing fragments to produce pBR322-CE16-HNA430T.

Before transfection, Hep-2 cells were seeded into six-well plates and grown to 80–90% confluence. The cells were then cotransfected with the full-length cDNA plasmid and pCAGGS-NP, pCAGGS-P, and pCAGGS-L at a ratio of 2 μg:1 μg:1 μg:0.5 μg by using Lipofectamine 2000. Along with the transfection mixture, 1 focus-forming unit per cell of MVA/T7 expressing T7 RNA polymerase was added. The plate was incubated at 37 °C for 24 h with Opti-MEM, and then the medium was replaced with DMEM with 2% FBS. After 3 days of incubation, the cells were harvested and inoculated into 9-day-old SPF chicken embryos to rescue the viruses. After 3 days, the allantoic fluid was harvested and checked by using a haemagglutination (HA) assay [17]. Viral RNA was extracted from HA-positive allantoic fluid according to the manufacturer’s protocol supplied with a StarSpin Animal RNA Mini Kit (Genstar, Beijing, China), and cDNA was synthesized from viral RNA using a reverse transcription PCR kit (Genstar, Beijing, China). The genome was sequenced by Sanger sequencing with three independent parallel tests to confirm the presence of the desired mutations.

### Indirect immunofluorescence assay (IIFA)

To observe the expression of HN protein, 1 μg of original HNs or mutant HNs was transfected into BHK-21 cells by using Lipofectamine 2000 Transfectant Reagent (Invitrogen, CA, USA). At 24 h post-transfection, each well was fixed with PBS containing 4% paraformaldehyde and blocked with PBS containing 1% bovine serum albumin (BSA). Then, the cells were incubated with an HN monoclonal antibody made by our laboratory at an optimized dilution of 1:1000 and incubated with Alexa Fluor^®^ 594-conjugated goat anti-mouse IgG (H + L) at a 1:500 dilution (Abcam, Shanghai, China) as the secondary antibody. Photographs of the cells were recorded under a fluorescence microscope (IX73; OLYMPUS, Tokyo, Japan). Empty cells were used as mock controls, and empty pCAGGS was used as the negative control. For rescued viruses, BHK-21 cells were incubated with viruses at an MOI of 0.1 for 12 h or 24 h. An IIFA at the virus level was performed with the methods described above.

### Flow cytometry (FCM) assay

To quantitate the cell surface expression of the four HNs, 1 μg of original HNs or mutant HNs was transfected into BHK-21 cells by using Lipofectamine 2000. At 24 h post-transfection, the cells were treated with 50 mM EDTA-PBS solution and blocked with PBS containing 1% BSA. Then, the cells were incubated with an HN monoclonal antibody at a dilution of 1:1000 and incubated with allophycocyanin (APC)-conjugated goat anti-rat IgG at a 1:500 dilution (Beyotime, Shanghai, China). After being washed twice, the cells were fixed with PBS containing 4% paraformaldehyde, and the mean cell surface fluorescence intensity was detected by using a flow cytometer (BD Biosciences, San Jose, CA, USA).

### Haemadsorption (HAd) assay

BHK-21 cells were transfected with 1 µg of original HNs or mutant HNs for 16 h, the medium was removed, and the cells were washed with precooled PBS. Then, 1 mL of 2% chicken red blood cell (CRBC) suspension was added to each well. After incubation on ice for 30 min, 100 µL of 50 mM NH_4_Cl solution was added to lyse the CRBCs bound to cells infected by the virus, and the mixture was collected and centrifuged (10 000 *g*, 4 °C, 5 min). Then, 100 µL of each supernatant was aspirated into a 96-well plate. The absorbance was measured at a wavelength of 540 nm using an ELISA reader (Epoch; BioTek, Winooski, VT, USA). For rescued viruses, BHK-21 cells were incubated with viruses at an MOI of 1 for 8 h. HAd activity at the virus level was determined by the methods described above.

### NA assay

BHK-21 cells were transfected with 1 µg of original HNs or mutant HNs for 16 h. Then, the cells were detached from the plates using 50 mM EDTA-PBS. After being washed twice with cold PBS and centrifuged to remove the supernatants, the cells were freeze–thawed 3 times at −80 °C with 20 µL of radioimmunoprecipitation assay buffer (Solarbio, Beijing, China). After centrifugation (10 000 *g*, 4 °C, 5 min), 10 µL of supernatant was used to detect NA activity with an NA assay kit (Beyotime, Shanghai, China) and a microplate reader (Spark; TECAN Group AG, Männedorf, Switzerland). For rescued viruses, BHK-21 cells were incubated with viruses at an MOI of 1 for 8 h. NA activity at the virus level was determined by the methods described above.

### Plaque formation assay

BHK-21 cells were cotransfected with 0.5 µg each of HNs and F plasmid. After 24 h, the fusion regions were randomly photographed using an inverted microscope, and the plaque sizes were determined using ImageJ software to compare the fusion-promoting activity of the HN proteins. For rescued viruses, BHK-21 cells were incubated with serially diluted virus for 24 h, and the supernatants were replaced with DMEM containing 1% methylcellulose (Solarbio, Beijing, China). After 3 days, the cells were fixed with 4% formaldehyde and stained with 1% crystal violet. The sizes of the plaques formed by the 2 viruses in BHK-21 cells were evaluated.

### Western blot analysis

Western blotting was used to detect the cleavage promotion of HN proteins. BHK-21 cells were cotransfected with 1 µg each of HNs and F plasmid. After 24 h, the total protein was extracted from cells with 50 mM EDTA. The cells were pelleted, washed, and lysed. Then, the polypeptides were analysed by SDS–PAGE. An HN monoclonal antibody (1:3000) or HA-labelled specific mouse primary antibody (1:3000; Invitrogen, Carlsbad, CA, USA) and a secondary antibody (HRP-conjugated goat anti-mouse IgG, 1:3000; Invitrogen, Carlsbad, CA, USA) were used. The protein bands were exposed with a chemiluminescence imager (MiniChemi610; Sagecreation, Beijing, China). The protein load was normalized to the GAPDH signal (1:3000; Sungene Biotech, Tianjin Binhai New Area, China). The western blots were quantified by the F1/F0 densitometry ratio using ImageJ software.

### Growth kinetics

The growth dynamics of rescued viruses were determined in DF-1 cells using multicycle growth conditions. Cells in 6-well culture plates were infected with viruses at an MOI of 0.01. After 1 h of adsorption, the cells were incubated with DMEM containing 1% FBS. The culture supernatants were collected at 12 h intervals until 60 h with an equal volume of culture medium. The viral titres in the collected supernatants of BHK-21 cells were determined via a plaque formation assay.

### Pathogenicity index

The pathogenicity of the rescued viruses was determined by MDT tests in 10-day-old SPF embryonated chicken eggs and ICPI tests in 1-day-old SPF chicks according to standard assay methods [18]. The pathotype definitions determined by the MDT were velogenic (< 60 h), mesogenic (60–90 hl), and lentogenic (>90 h), and those determined by the ICPI were velogenic (0.70–1.50), mesogenic (1.50–2.00), and lentogenic (0.00–0.70).

### Pathogenicity assessment in chickens

To evaluate the pathogenicity of the rescued viruses in chickens, three groups of 4-week-old SPF chickens of 20 birds each (10 for tissue sampling and 10 for clinical observation) were inoculated with 10^5^ pfu/100 μL of the indicated virus or with 0.9% NaCl per bird via intraocular and intranasal routes. The birds were monitored for clinical signs daily for 14 days post-infection (dpi) and scored as follows: 0 for normal health, 1 for illness, 2 for paralysis/torticollis/wing drop/incoordination, 3 for prostration, and 4 for death [19]. Three birds from each group were euthanized at 3 and 5 dpi for gross lesion observation, and tissue samples were separated into two parts. One part (including tissues from the brain, thymus, trachea, lungs, proventriculus, spleen, duodenum, pancreas, caecal tonsils, and bursa of Fabricius) was used for virus titration in BHK-21 cells. These tissue samples were homogenized in phosphate-buffered saline containing a final concentration of 10 000 units of penicillin G and streptomycin, and the viral titres in the collected supernatants were determined via 50% tissue culture infective dose (TCID_50_) assessment in BHK-21 cells using the Reed and Muench method [20]. The other part (including tissues from the brain, trachea, lungs, proventriculus, duodenum, pancreas, and bursa of Fabricius) was fixed in 10% neutral buffered formalin for histopathology. The fixed tissues were routinely embedded in paraffin, sectioned, stained with haematoxylin–eosin, and examined for lesions using light microscopy. Necropsies were carried out, and external and internal abnormalities were recorded.

### Statistical analysis

All significant differences were determined using GraphPad Prism Software (version 8.0, California San Diego, CA, USA). The experimental data are expressed as the mean ± standard deviation (SD). A nonparametric (chi-square) test was applied for viral loading in tissues. ANOVA was employed for multiple comparisons of experimental groups and was corrected by the Bonferroni post test to determine significance. Statistical significance was set according to *P* values calculated based on two-tailed unpaired t tests (95% confidence levels). *p < 0.05; **p < 0.01; ***p < 0.001.

## Results

### Cell surface expression of the original and mutant HNs

**Figure 2:**
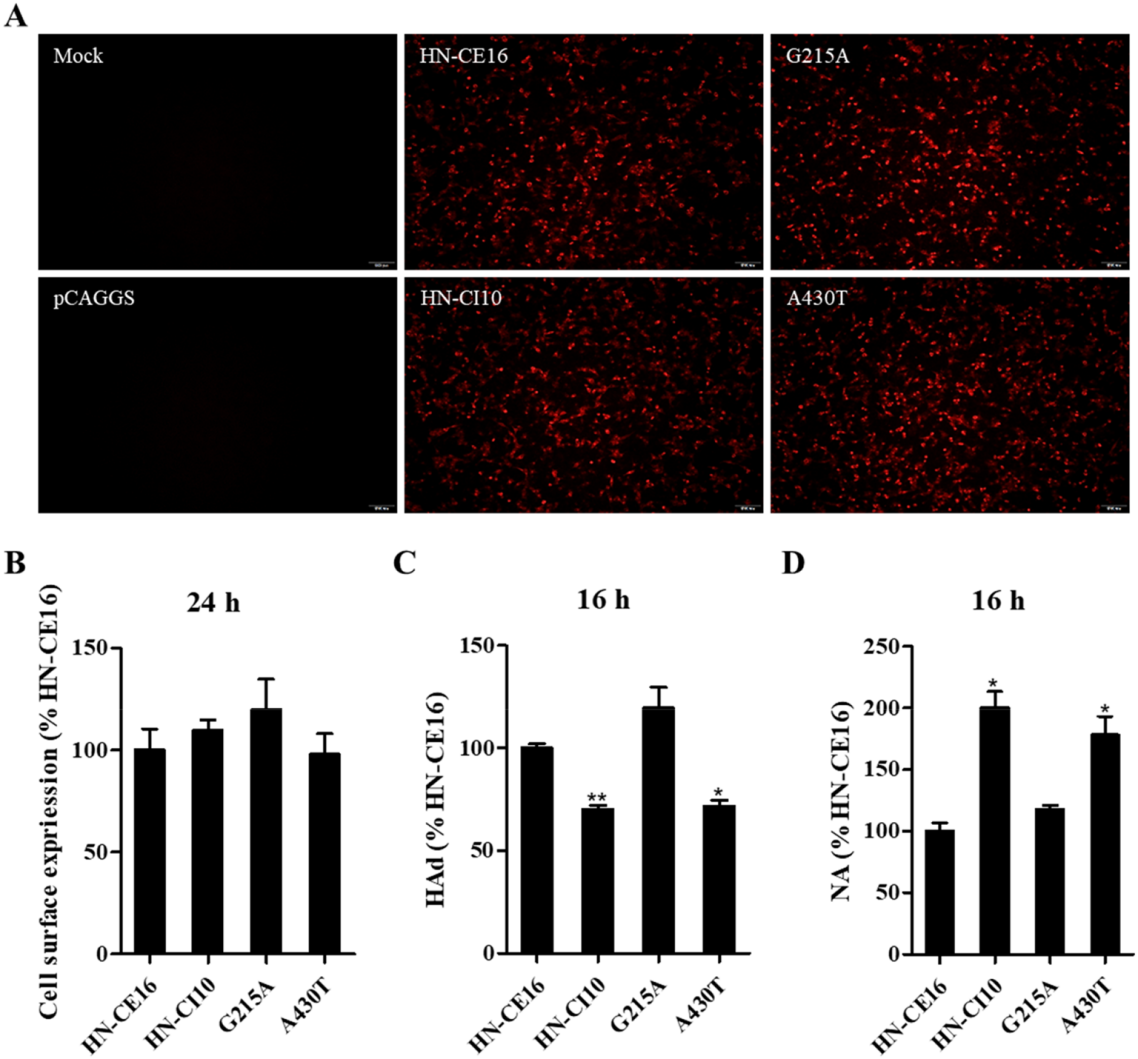
**Biological activities of the original HNs.** The expression of each HN protein at the cell surface was determined by **A** IIFA and FCM **B** analysis at 24 h post-transfection of BHK-21 cells by using a primary HN monoclonal antibody and a fluorescein-labelled secondary antibody. Empty cells were used as mock cells, and empty pCAGGS was used as the negative control. **C** The HAd activity was determined based on the ability of HN expressed at the cell surface at 16 h post-transfection to adsorb chicken erythrocytes at 4 °C. **D** NA activity was determined as the ability of the cell surface HN proteins to catalyse the release of sialic acid at 16 h post-transfection. All marks indicate significance in comparison to HN-CE16 (100%), and the results are presented as the mean ± SD of the results of three independent experiments. *p < 0.05, **p < 0.01.

An IIFA and FCM were used to determine whether these HN proteins differed in expression at the cell surface. The IIFA results showed that the four HNs were all efficiently expressed in BHK-21 cells (Figure 2A). Then, the cell surface expression of these HNs was quantitated by FCM (Figure 2B). The HN-CI10, G215A, and A430T protein expression levels at the cell surface were not significantly different from the HN-CE16 protein expression levels, suggesting that these HN proteins were not misfolded.

### Biological functions of the original HNs and mutant HNs

HAd and NA assays were used to determine the activity of receptor recognition and release of sialic acid, respectively (Figures 2C, D). The results showed that HN-CI10 and A430T significantly weakened the HAd to 70.1% and 72.5% but increased the NA activity to 195.5% and 178.3% of that of HN-CE16, respectively. G215A enhanced the HAd and NA activity to 119.6% and 117.9% of that of HN-CE16, respectively, which did not cause noticeable differences in the biological functions of the HN protein. In summary, the A430T mutation impaired receptor recognition activity but significantly improved the release of sialic acid from the substrate of HN.

### Promotion of the fusion activity of F by HNs

**Figure 3:**
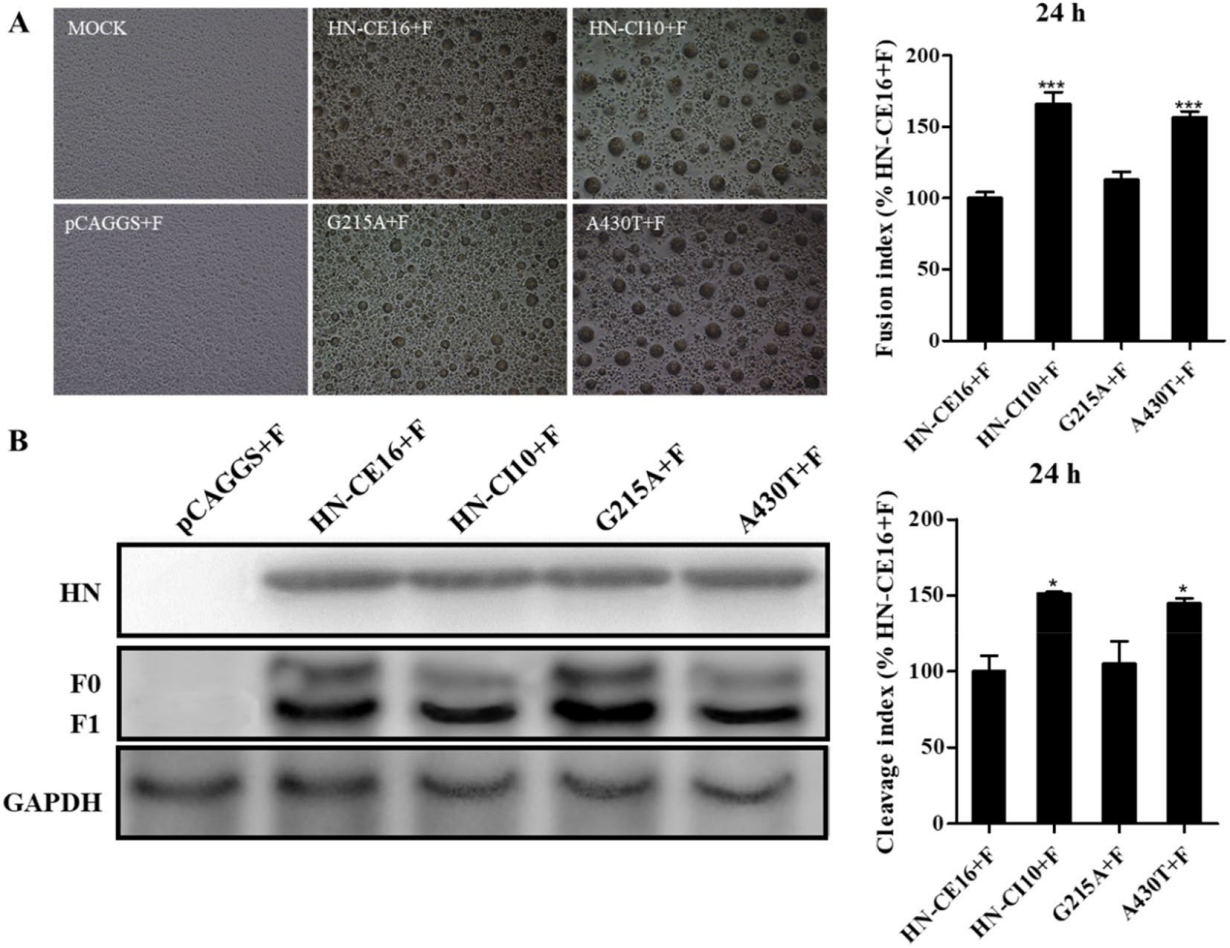
**Syncytium formation and protein expression in BHK-21 monolayer cells coexpressing HN and F proteins for 24 h.** **A** The extent of syncytium formation is shown for representative BHK-21 monolayer cells alone or cells expressing pCAGGS + F, HN-CE16 + F, HN-CI10 + F, or HN bearing the G215A and A430T substitutions. The cells were photographed under a fluorescence inverted microscope with 100-fold magnification, Bar = 100 µm. The fusion indices were determined by measuring the syncytium diameters (*n* = 10). **B** Total HN and F protein expression was verified by western blotting. The cleavage indices were determined according to the F1/F0 ratios, which were quantified by densitometry using ImageJ software. All marks indicate significance in comparison to HN-CE16 (100%), and the results are presented the mean ± SD of the results of three independent experiments. *p < 0.05, ***p < 0.001.

Membrane fusion assays and Western blotting were used to assess the fusion-promoting activity and cleavage-promoting activity of the F protein by the HN proteins. According to the index of fusion-promoting activity, the syncytia formed by HN-CI10 (166.3%) and A430T (156.6%) were significantly larger than those formed by HN-CE16 (100%) (Figure 3A). According to the F1/F0 ratios, the cleavage ability of the F protein promoted by HN-CI10 (151.4%) and A430T (140.9%) was significantly better than that promoted by HN-CE16 (100%) (Figure 3B). However, G215A did not cause apparent differences in either the fusion-promoting activity or cleavage-promoting activity of the F protein. Thus, the protein level results indicated that the HN430 aa site was a key site for fusion activity.

### Recovery of rCE16 and rCE16-HNA430T

To further assess the influence of the A430T mutation on viral pathogenicity, plasmid-based reverse genetics was used to rescue the original virus rCE16 and the mutant virus bearing A430T (rCE16-HNA430T). To examine genetic stability, the recovered viruses were serially propagated for 10 passages in 10-day-old embryos, and their whole genomes were sequenced after the third, fifth, and tenth passages. The results indicated that rCE16 and rCE16-HNA430T were stable and that no other variations were present in the whole genome.

### Biological characteristics of the chimeric NDVs

**Figure 4:**
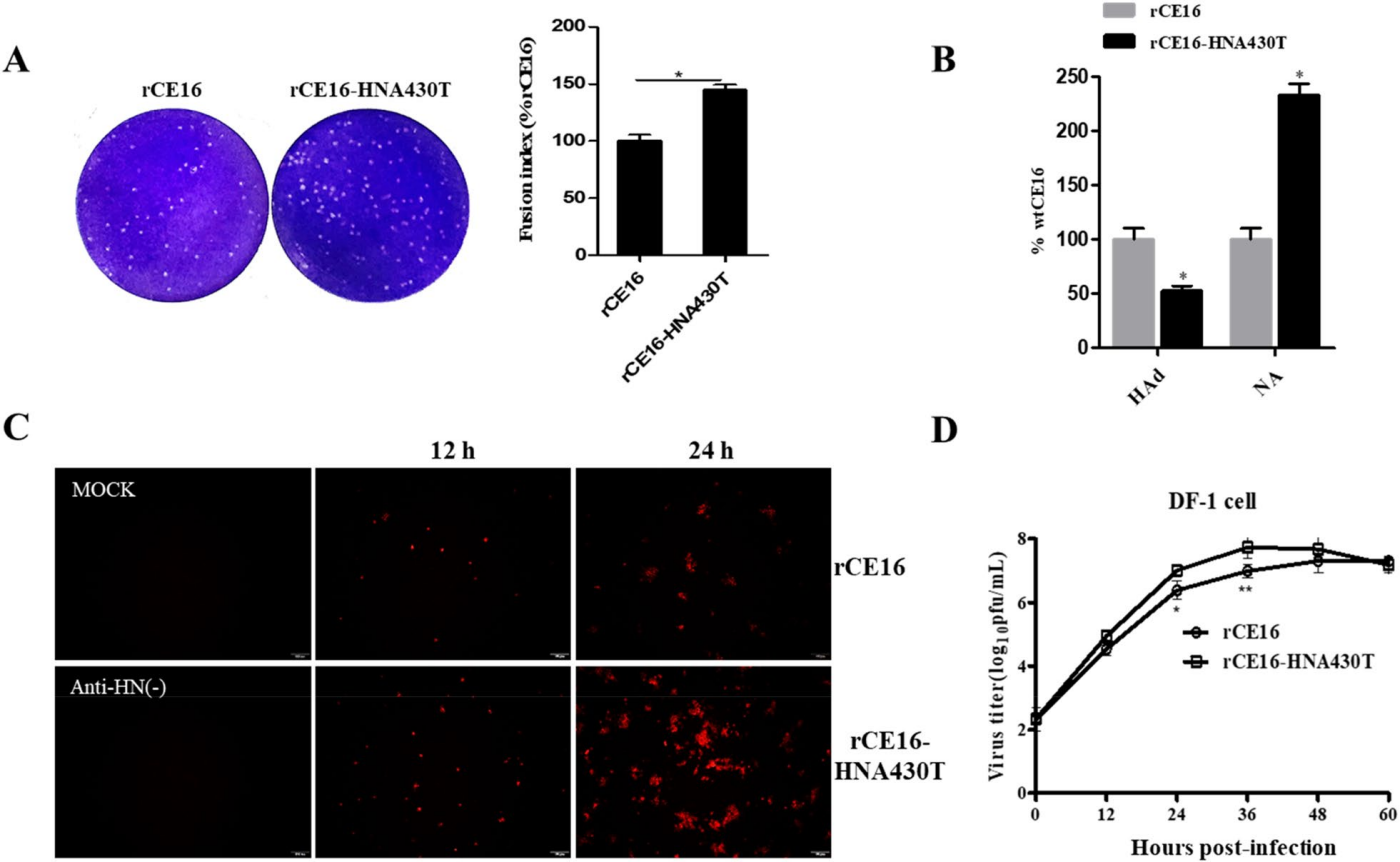
**Biological characteristics of rescued rCE16-HNA430T and rCE16.** **A** Representative plaque formation induced by viral infection of BHK-21 cells. Monolayer cells were fixed with 4% paraformaldehyde and stained with crystal violet. The fusion indices were determined by measuring the plaque diameters (*n* = 10). **B** The relative HAd ability and NA activity were assessed for 1 MOI virus-infected BHK-21 cells. **C** The expression of HN proteins was detected by IIFA. BHK-21 cells were infected with virus at an MOI of 0.01 for 24 h. The cells were then incubated with a primary HN monoclonal antibody and a fluorescein-labelled secondary antibody and then photographed under a fluorescence inverted microscope with 100-fold magnification, Bar = 100 µm. The mock cells were empty cells incubated with the primary antibody and secondary antibody. Anti-HN(–) indicates infected cells incubated only with the secondary antibody. **D** Growth kinetics of rCE16-HNA430T and rCE16 in DF-1 cells treated with an MOI of 0.01. All marks indicate significance in comparison to rCE16 (100%), and the results are presented as the mean ± SD of the results of three independent experiments. *p < 0.05, **p < 0.01.

The cytopathic effects of the chimeric NDVs were evaluated by measuring the sizes of the plaques formed in BHK-21 cell monolayers. rCE16-HNA430T exhibited a plaque size approximately 50% larger than that of rCE16 (Figure 4A), indicating that the A430T mutation influences the fusion promotion activity of the HN protein.

To analyse whether the A430T mutation modulates the biological activities of HN in BHK-21 cells, HAd and NA tests were performed on the chimeric NDVs. Compared with those of rCE16, the HAd activity and NA activity of the mutant viruses were reduced by approximately 50% and significantly increased by 140%, respectively, consistent with the protein level results for the biological activities of HN (Figure 4B). These results suggest that the A430T mutation influences viral entry and release, which have combined effects on HN protein function alternations.

Next, the growth kinetics of the two strains were compared using an IIFA and multicycle growth curve analyses. The IIFA results showed that the viral titres of rCE16-HNA430T were higher than those of rCE16 at 12 h and 24 h. The plaques formed by rCE16-HNA430T were also obviously larger than those formed by rCE16 at 24 h, which was consistent with the increased plaque sizes in cells (Figure 4C). The growth kinetics results in DF-1 cells also showed that rCE16-HNA430T presented significantly higher viral titres at 24 and 36 h than rCE16 (*p* < 0.05). The HN-A430T mutant grew faster than rCE16 at 12–36 h but grew poorly between 36 and 60 h (Figure 4D).

### Pathogenicity of the chimeric NDVs

To understand whether the A430T mutation influences viral virulence, we compared the pathogenicity of the chimeric NDVs in chickens using the ICPI and MDT. The results showed that the ICPI and MDT of rCE16-HNA430T (1.38 and 87 h) were higher than those of rCE16 (0.34 and > 120 h). These data suggested that the A430T mutation enhanced pathogenicity in chickens.

### Pathogenicity of the chimeric NDVs in 4-week-old chickens

**Figure 5:**
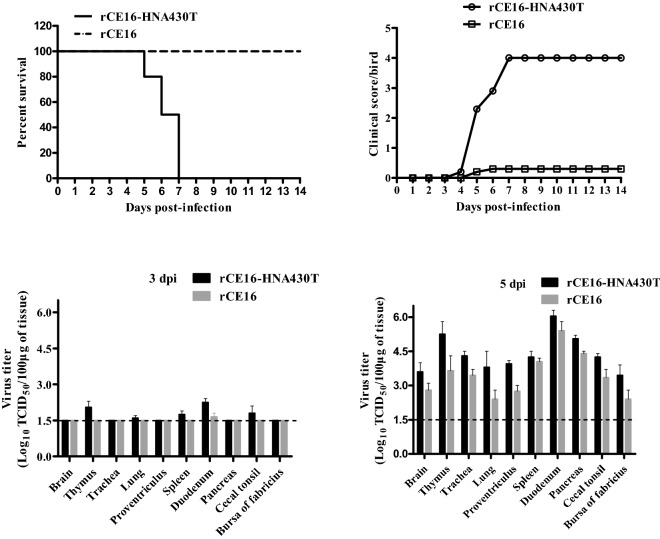
**Pathogenicity of rCE16-HNA430T and rCE16 in 4-week-old chickens.** Birds were inoculated with 10^5^ pfu/100 μL of the indicated virus with 0.9% NaCl per bird via the intraocular and intranasal routes. **A** Survival curves of 4-week-old chickens. The survival curves were compared using the log-rank test. The survival data were analysed using Prism 8.0 (GraphPad Software Inc., San Diego, CA, USA). **B** Clinical scores of 4-week-old chickens. The mean scores per group per day are shown. **C** Viral loads of rCE16-HNA430T and rCE16 in chickens. Three chickens of each virus-infected group were sacrificed at 3 and 5 dpi. The detection limit was 10^1.48^ TCID_50_/mL. A nonparametric (chi-square) test was applied. The values presented are the means ± SDs of the viral titres of the indicated viruses.

To further evaluate viral virulence, the pathogenicity and viral titres of the chimeric NDVs were compared in 4-week-old chickens. The results showed that the pathogenicity of the two viruses differed greatly in chickens. The chickens infected with rCE16-HNA430T first developed clinical signs at 4 dpi with slight depression (2/10) and developed severe depression at 5 dpi with prostration (3/10), wing drop/incoordination (4/10), and death (2/10); all died by 7 dpi (Figures 5A, B). At necropsy, all euthanized chickens showed slight haemorrhaging in the throat and trachea; atrophy of the thymus accompanied by haemorrhaging; severe haemorrhaging in the lungs, proventriculus, duodenum, pancreas, and caecal tonsils; and severe spleen swelling at 3–5 dpi. In comparison, the birds infected with rCE16 developed slight depression at 5 dpi, but all survived (Figures 5A, B). At necropsy, all euthanized chickens showed slight punctate haemorrhaging in the thymus, lungs, pancreas, duodenum, and caecal tonsils and less severe spleen swelling at 5 dpi. Additionally, no clinical signs or lesions were observed in the control group.

The viral titre results (Figures 5C, D) showed that both chimeric NDVs could replicate in all sampled tissues and that the viral titres in the sampled tissues gradually increased over time. The viral titres in the tissues of the birds infected with rCE16-HNA430T were slightly higher than those in the tissues of the birds infected with rCE16 at 3 and 5 dpi, which was consistent with the growth kinetics in cells.

Histopathological analysis (Additional file 4) revealed that chickens infected with rCE16-HNA430T exhibited severe tissue pathological changes in all of the sampled tissues: cellular atrophy and nuclear disappearance forming red clumps (arrow 1) as well as vasoconstriction (arrow 2) in the cerebrum; severe epithelial necrosis (arrow 1) and lymphocyte infiltration (arrow 2) in the trachea; severe mucosal epithelial necrosis and shedding (arrow 1) and capillary congestion of the lamina propria and muscularis (arrow 2) in the proventriculus; severe intestinal villus necrosis, shedding (arrow 1), and capillary congestion (arrow 2); recess cell degeneration (arrow 3) in the duodenum; and acinar cell nuclear concentration and disappearance (arrow 1) and capillary congestion (arrow 2) in the pancreas. For rCE16, mild tissue pathological changes were observed in sampled tissues: slight epithelial necrosis and shedding (arrow 1) in the trachea; recess cell necrosis (arrow 1) and capillary congestion of the lamina propria and muscularis (arrow 2) in the proventriculus; and acinar cell atrophy and disappearance (arrow 1) and capillary congestion (arrow 2) in the pancreas.

## Discussion

In this study, G215A and A430T mutations were introduced into the HN gene by reverse mutation from CE16. The effects of the mutations were estimated on two levels: the protein and virus levels. At the protein level, A430T had considerable influence on HN functions, supporting the idea that HN430 is an important aa site for NDV. The reverse genetics system is an effective molecular tool that has enabled the roles of viral proteins in viral virulence to be determined by genetic approaches [7, 21, 22]. Herein, the A430T chimeric virus was successfully recovered by reverse genetics to investigate the effect of the HN430 substitution on viral activities. The results showed that the A430T mutation exhibited markedly enhanced pathogenicity and fusion promotion activity and slightly promoted viral titres. Thus, our study authenticates a new virulence site that has not yet been reported.

The HN protein of NDV has been recognized to promote membrane fusion by interacting with the F protein [7–9]. In this study, the A430T-containing HN protein promoted membrane fusion activity in cells, and the A430T chimeric virus also increased the syncytial number and size. Furthermore, western blotting also revealed that the A430T-containing HN protein had an obvious effect on promotion of F protein cleavage. All this evidence indicates that the aa residue at the HN430 site can influence fusion ability by promoting cleavage of the F protein. In general, the stalk (83–114 aa) of the HN protein has been confirmed to play roles in HN-F interactions [11–13]. However, the HN430 aa site is located in the globular head of HN, showing that mutations in other regions may have some effects on F protein cleavage. This phenomenon has also been observed in previous studies. Li et al. constructed several function-deficient mutants with mutations in the globular domain of HN that exhibited fusion deficiency and failed to interact with F at the cell surface, as shown proven by coimmunoprecipitation assays [22].

Interestingly, the results also revealed some correlations between HN functions and viral replication in cells and chickens, which has also been reported previously [14, 23]. Analyses of growth kinetics in cells and viral titres in tissue samples of chickens showed that rCE16-HNA430T had higher levels of viral replication than rCE16. HN is mainly responsible for receptor recognition, cell fusion promotion, and NA activity, which has no direct correlation with virus replication [11–13]. Here, the A430T mutant dramatically facilitated NA activity. The NA activity of the HN protein acts to hydrolyse sialic acid to release progeny virions [13]. Thus, we speculate that the higher viral titres of the A430T chimeric virus might have been attributable to the increased NA activity, which induced the release of more progeny virions and further resulted in enhanced pathogenicity.

## Supplementary Information

**Additional file 1.** **Primers used for the construction of F and HN.**

**Additional file 2.** **Primers used for the construction of CE16 helper plasmids.**

**Additional file 3.** **Primers used during full-length cDNA synthesis.**

**Additional file 4.** **Histopathology of tissue samples of 4-week-old chickens infected with rCE16-HNA430T and rCE16.** Three birds were sacrificed at 5 days post-inoculation, and the tissues were fixed with formalin, sectioned, and stained with haematoxylin and eosin. Cells were photographed under a fluorescence inverted microscope at 200-fold magnification, Bar = 50 µm.

## Data Availability

All data generated or analysed during this study are included in this published article and its additional information files.
