# Supplementary material for: Identification of a new amino acid mutation in the HN protein of NDV involved in pathogenicity
Source: Vet Res. 2021 Dec 20;52:147. doi: 10.1186/s13567-021-01019-4 (PMC8686287; doi:10.1186/s13567-021-01019-4)
Supplement: Supplementary file 1 — Additional file 1. Primers used for the construction of F and HN. [file 13567_2021_1019_MOESM1_ESM.docx]

| cDNA  fragments | Primers | Enzyme |
| --- | --- | --- |
| 1 | F-Fw: CGgaattcATG*GATTACAAGGACGATGACGATAAG*GGCTCCAAACCTTCCAC  F-Rev: CCGctcgagTCATGTTCTTGTAGTGGCT | *EcoRI*  *XhoI* |
| 2 | HN-Fw: CCatcgatATGGACCGTGTAGTCAGCAG  HN-Rev: CCGctcgagTCAAACTCTATCATCTTTAAG | *ClaI*  *XhoI* |
| 3 | HNG215A-Rev: CTTCCCTGTTgCAGATGTCCG  HNG215A-Fw: CGGACATCTGcAACAGGGAAG |  |
| 4 | HNA430T-Rev: GTTATGGACTGtCATAGGGTAT  HNA430T-Fw: ATACCCTATGaCAGTCCATAAC |  |

Note: Fw represents forward, Rev represents reverse. Virus-specific sequences are underlined, and restriction sites are shown in lowercase. Mutation sites are shown in lowercase and marked as red. Flag tag are italic.
