# Supplementary material for: Identification of a new amino acid mutation in the HN protein of NDV involved in pathogenicity
Source: Vet Res. 2021 Dec 20;52:147. doi: 10.1186/s13567-021-01019-4 (PMC8686287; doi:10.1186/s13567-021-01019-4)
Supplement: Supplementary file 2 — Additional file 2. Primers used for the construction of CE16 helper plasmids. [file 13567_2021_1019_MOESM2_ESM.docx]

| cDNA fragments | Primers | Enzyme |
| --- | --- | --- |
| 1 | NP-Fw： CGgaattcATGTCGTCCGTCTTCGACGAGTACG  NP-Rev： CCGctcgagTCAGTACCCCCAATCAGTGTCATTGT | *EcoRI*  *XhoI* |
| 2 | P-Fw： CGgaattcATGGCCACTTTTACAGATGCTGAG  P-Rev： CCGctcgagTCAACCATTCAGCGCAAGGCGTTTGA | *EcoRI*  *XhoI* |
| 3 | L-Fw： CATGccatggATGGCGGGCTCCGGTCCCGAAAG  L-Rev： CGggatccTTATGGGTCATTGTTACTGTAATATC | *NcoI*  *BamHI* |

Note. Fw represents forward, Rev represents reverse. Virus-specific sequences are underlined, and restriction sites are shown in lowercase.
