# Supplementary material for: Identification of a new amino acid mutation in the HN protein of NDV involved in pathogenicity
Source: Vet Res. 2021 Dec 20;52:147. doi: 10.1186/s13567-021-01019-4 (PMC8686287; doi:10.1186/s13567-021-01019-4)
Supplement: Supplementary file 3 — Additional file 3. Primers used during full-length cDNA synthesis. [file 13567_2021_1019_MOESM3_ESM.docx]

| cDNA  fragments | Primers | Enzyme | Order of  cloning |
| --- | --- | --- | --- |
| 1 | Fw： AGTTggcgcgcc*TAATACGACTCACTATAGGG*ACCAAACAGAGAATCTGTGAGGTACGATAAAAGG  Rev： CCGGCGCGCCgtttaaacTGTTGGTGGACCGAGTCGTGCCCGTGTTG | *Asc I*  *Pme I* | 8 |
| 2 | Fw： AGCTTTgtttaaacAGAACCTGAGACATTAGAAAAAAATACG  Rev： TGCCttaattaaGGTTGTGCGATCATTCGGGGGAGTGGG | *Pme I*  *Pac I* | 7 |
| 3 | Fw： TGCCttaattaaCCCAACAGAATCAGAGATTAAGAAAAAATACGG  Rev： ACGATTgcgatcgcGATAGATCGTTGCCTAGTGTCGCGATG | *Pac I*  *AsiS I* | 6 |
| 4 | Fw： ACGATTgcgatcgcTTATAGGTAGTTCACCTGTCTATC  Rev： ATAGTTTAgcggccgcAATTGACACATAGGTTACTATTGAGGATAC | *AsiS I*  *Not I* | 5 |
| 5 | Fw： ATAAGAATgcggccgcCTGTTCAACAGAAGATTTTAGAAAAAAATACCAG  Rev： ATCAGGtacgtaTGTTTTTTCTTAATAAAGTGACTATGAATAAG | *Not I*  SnaB I | 4 |
| 6 | Fw： CATGCCATGGtacgtaGTAGTGAGATCTAAGAGAAAACAAC  Rev： TCCccgcggGTGAATCGCCTCTTGATTGAGTAAGAATTCAG | *SnaB I*  *Sac II* | 3 |
| 7 | Fw： TCCccgcggGTCGCACATGCTATCATGGAGGCAAG  Rev： GCtctagaCATGTAATATAGATTAGCTGGGAAC | *Sac II*  Xba I | 2 |
| 8 | Fw： GCtctagaAAGAGCCTTAATTTAATCAGGGAACGAG  Rev： GGTCcggaccg*CGAGGAGGTGGAGATGCCATGCCGACCC*ACCAAACAAAGATTTGGTGAATGACAG | *Xba I*  *Rsr II* | 1 |

Note. Fw represents forward, Rev represents reverse. T7 RNA polymerase promoter sequences in segment 1 Fw and the partial HDV ribozyme sequence (24-nt) in segment 8 Rev are marked in italic type, the virus-specific sequences are underlined, and restriction sites are shown in lowercase.
