# Supplementary material for: Identification of a new amino acid mutation in the HN protein of NDV involved in pathogenicity
Source: Vet Res. 2021 Dec 20;52:147. doi: 10.1186/s13567-021-01019-4 (PMC8686287; doi:10.1186/s13567-021-01019-4)
Supplement: Supplementary file 4 — Additional file 4. Histopathology of tissue samples of 4-week-old chickens infected with rCE16-HNA430T and rCE16. Three birds were sacrificed at 5 days post-inoculation, and the tissues were fixed with formalin, sectioned, and stained with haematoxylin and eosin. Cells were photographed under a fluorescence inverted microscope at 200-fold magnification, Bar = 50 µm. [file 13567_2021_1019_MOESM4_ESM.docx]

**Proventriculus**

**Control**

**Brain**

**Trachea**

**rCE16**

**rCE16-HNA430T**

**Duodenum**

**Pancreas**

**2**

**1**

**1**

**2**

**1**

**1**

**1**

**2**

**2**

**3**

**2**

**2**

**2**

**1**

**1**

**3**

**1**

**2**

**1**

**2**

**2**
